# Pragmatic implementation of intravascular lithotripsy in Japan based on the guidelines for proper use

**DOI:** 10.1007/s12928-025-01224-4

**Published:** 2025-12-03

**Authors:** Takeshi Shiba, Masato Nakamura, Ken Kozuma, Teruyasu Sugano, Koichi Aizawa, Naoyuki Yabana, Kensuke Ishii

**Affiliations:** 1 Pharmaceuticals and Medical Devices Agency, Tokyo, Japan; 2 Division of Minimally Invasive Treatment in Cardiovascular Medicine, Toho University Ohashi Medical Center, Tokyo, Japan; 3 Department of Cardiology, Teikyo University Hospital, Tokyo, Japan; 4 Department of Cardiology, Saiseikai Yokohamashi Nanbu Hospital, Yokohama, Kanagawa Japan; 5 Office of Medical Devices I, Pharmaceuticals and Medical Devices Agency, 3-2-2 Kasumigaseki, Chiyoda-ku, Tokyo, 100-0013 Japan

**Keywords:** Guidelines for proper use, Intravascular lithotripsy, Percutaneous coronary intervention

## Abstract

The Shockwave C^2^ Coronary Intravascular Lithotripsy (IVL) system (Shockwave Medical, Santa Clara, California), designed to disrupt calcified, *de novo* coronary lesions, was approved for use in Japan in 2022. Unlike in other countries, where IVL is limited to prestenting use, Japan permitted its broader application, owing to the unique percutaneous coronary intervention environment in this country. Moreover, given that severely calcified lesions in coronary arteries are relatively difficult to treat and that treatment strategies vary widely, clarifying the position of IVL and establishing an algorithm for treating calcified lesions based on available evidence would be helpful in daily clinical practice. Additionally, not all indications for IVL use are supported by evidence; thus, the algorithm should be modified according to the accumulated evidence. To ensure postmarketing safety, it was introduced in stages, in cooperation with relevant academic societies. The guidelines for proper IVL use were established by the academic society. The use of concomitant procedures was restricted during the initial phase of IVL introduction while the guidelines were revised in response to postmarket clinical evidence. The Dual-Prep Registry, designed to evaluate the safety and effectiveness of IVL following atherectomy in severely calcified coronary lesions, was conducted at 20 sites involving 118 patients with 120 lesions. The 30-day major adverse cardiac event-free rate, the primary safety endpoint, was 98.3%, and the procedural success rate, the primary efficacy endpoint, was 98.3%. In 2025, based on the Dual-Prep Registry, the guidelines for proper IVL use were revised to address the combined use of an atherectomy device. Thus, this review demonstrates pragmatic efforts to introduce medical devices in Japan, utilizing the guidelines for proper use.

## Introduction

Medical devices evolve rapidly; thus, their appropriate use may change based on clinical evidence. Hence, rebalancing between premarketing and postmarketing may be crucial and useful for the approval of their use. To restore the balance and to introduce innovative medical devices appropriately into the medical setting, establishing guidelines for proper use in cooperation with relevant academic societies and planning training programs for physicians have been considered important. Opportunities for such initiatives have been increasing in recent years. Guidelines for proper use play a significant role in medical device utilization, owing not only to the device’s performance but also the wide-ranging factors that affect treatment outcomes. Such factors include the type of patients, the type of facility and physicians, and the manner of using the device. Generally, premarket clinical trials are conducted on specific types of patients and lesions to test the safety and effectiveness of the device. However, real-world data are important to confirm the device’s usefulness, especially for innovative devices, considering that these trials do not include all patient types or situations and that the experience of using the device is still limited, even in other countries. The roles of postmarket surveillance and postmarket study are growing, and the collection and establishment of evidence are becoming increasingly important [1].

In 2022, Japan approved the Shockwave C^2^ Coronary Intravascular Lithotripsy (IVL) system (Shockwave Medical, Santa Clara, California) for treating severely calcified lesions in coronary arteries [2]. In other countries; IVL is limited to prestenting use. However, considering the percutaneous coronary intervention (PCI) environment in Japan, it may be better to apply IVL for broader indications. Additionally, given that severely calcified lesions in coronary arteries is relatively challenging to treat and that treatment strategies vary widely, the applicant needs to cooperate with the Japanese Association of Cardiovascular Intervention and Therapeutics (CVIT) to implement postmarket safety measures for the safe introduction of IVL into clinical practice. CVIT has established evidence-based guidelines for the appropriate use of IVL, including a treatment algorithm. These guidelines are based on the severity of calcification as assessed by intravascular imaging evaluation and define treatment strategies accordingly. They also reflect the standard Japanese PCI practices, where imaging is routinely used both before and during PCI procedures. Furthermore, the guidelines advise against the use of IVL in procedures with insufficient evidence, such as that involving combination therapy with other products (e.g., atherectomy or drug-coated balloons [DCBs]). Such applications are not recommended at this stage and should only be considered following the accumulation of clinical evidence from planned studies. The efficacy and safety of combining IVL and atherectomy were evaluated through the Dual-Prep Registry. In 2025, CVIT’s guidelines for proper IVL use were revised according to the registry’s findings.

This case is a practical initiative to revise and optimize the guidelines for proper IVL use in clinical settings by collecting and evaluating real-world evidence in clinical practice following market approval, while limiting the scope of use during introduction, taking into consideration the product characteristics, the Japanese medical environment, and the concept of pre- and postmarketing rebalancing. This paper reviews the collaboration among industry, academia, and government and explores strategies to optimize the introduction of innovative medical devices in Japan. This review aimed to summarize the process of introducing IVL in Japan and was not intended for promotional purposes. The views expressed in this article are those of the authors and do not necessarily reflect those of the Pharmaceuticals and Medical Devices Agency (PMDA).

## Regulatory approval review of efficacy and safety of the IVL

### Disrupt CAD Ⅲ study and disrupt CAD Ⅳ study

The IVL system, comprising the IVL catheter and the IVL generator, is intended for disrupting calcified, *de novo* coronary lesions. The IVL catheter contains two emitter stations with two integrated lithotripsy emitters to deliver acoustic pressure pulses in the balloon. Electrical energy in the form of direct-current pulses delivered from the IVL generator to the lithotripsy emitters is converted into acoustic pressure pulses, which are then delivered to the target lesion through the balloon to disrupt the calcified lesion. The applicant submitted the results of the “Disrupt CAD III study,” a clinical study in the United States and Europe, and the “Disrupt CAD IV study,” a clinical study in Japan [3–6].

The Disrupt CAD III study was a multicenter, prospective, single-arm study that evaluated the efficacy and safety of the IVL System in treating severely calcified, *de novo* coronary lesions before stenting. The primary safety endpoint, defined as the 30-day major adverse cardiovascular event (MACE)-free rate, was 92.2% (353 of 383 participants; the lower bound of the one-sided 95% confidence interval [CI]: 89.9%), which exceeded the prespecified performance goal of 84.4%. The primary efficacy endpoint, defined as the procedural success rate (measured by stent delivery with a residual in-stent stenosis < 50% [as assessed by a core laboratory] and without in-hospital MACE), was 92.4% (355 of 384 participants; the lower bound of the one-sided 95% CI: 90.2%), also exceeding the prespecified performance goal of 83.4%.

The Disrupt CAD IV study was a multicenter, prospective, single-arm study in Japan, enrolling participants according to eligibility criteria similar to those used in the Disrupt CAD III study. This study aimed to verify the applicability of data extrapolated from the Disrupt CAD III study to the Japanese population. The primary safety endpoint, defined as the 30-day MACE-free rate, was 93.8% (60 of 64 participants), which was not inferior to that of the matched Disrupt CAD III study cohort (91.2%, 291 of 319 subjects). The primary efficacy endpoint, defined as the procedural success rate (measured by successful stent delivery with a residual in-stent stenosis < 50% [as assessed by a core laboratory] and without in-hospital MACE), was 93.8% (60 of 64 subjects), demonstrating noninferiority compared with the matched Disrupt CAD III study cohort (91.6%, 293 of 320 subjects).

### Basis of regulatory approval of the indication for IVL in Japan

The Disrupt CAD III and Disrupt CAD IV studies confirmed that the IVL system is effective and safe for treating severely calcified, *de novo* coronary lesions prior to stenting. Currently, a drug-eluting stent (DES) is the first-line option for PCI in Japan [7]. However, the stentless technique using a drug-coated balloon (DCB) is also accepted for treating certain lesions, such as those in small vessels or bifurcated sites. Therefore, the efficacy and safety of post-IVL treatment options other than stenting were reviewed.

- According to several publications [8–12], patients with calcified coronary arteries treated with DCB with and without atherectomy in Japan showed no serious angiographic complications with poor prognosis, suggesting that DCB is useful in patients ineligible for DES placement.
- In a pooled data analysis involving 628 participants (72 sites in 12 countries) from the Disrupt CAD I to IV studies, 561 participants were evaluated following IVL angiography. Treatment with the IVL system led to an increased minimum lumen diameter and a decreased percent diameter stenosis. Complications such as post-IVL perforation, abrupt closure, and no reflow were not reported. The incidence of dissections with blood flow restriction (Grade D or higher) and that of slow flow were as low as 1.8% and 0.4%, respectively [13]. In the ORBIT II Study of the “Diamondback 360 Coronary Orbital Atherectomy System,” the incidence of serious angiographic complications after atherectomy was 2.3% for severe dissection (Grade C or higher), 0.9% for perforation, 0.2% for slow flow, 0% for no reflow, and 0.9% for abrupt closure [14].
- Literature search on post-IVL treatments other than stenting found several publications regarding the combination of the IVL system and DCB [15–18]. None of these publications reported angiographic complications, including DCB-related thrombotic events. A follow-up for up to 15 months confirmed that the patients remained clinically stable.

These results are unlikely to suggest that post-IVL stentless treatment is associated with disease exacerbation as compared with the conventional procedures. Abrupt closure is a potential risk in stentless treatment; however, this risk can be mitigated by observing the blood vessel for severe dissection via intravascular ultrasound or optical coherence tomography (OCT), as with the conventional procedures. Two perforation cases in the Disrupt CAD III study occurred after stenting. Considering this, along with the insights from the Expert Discussion, treating physicians should have the discretion to select appropriate post-IVL treatment options. Ultimately, a general agreement was made, that is, the indication for the IVL system in Japan, unlike in other countries, should not be limited to the prestenting use (Table 1).

**Table 1:** Approval status of the IVL system in Europe, united States, and Japan

Country	Date of approval	Intended use or indication of the IVL system
Europe	June 2018	For lithotripsy-enhanced, low-pressure balloon dilatation of calcified, stenotic de novo coronary arteries prior to stenting.
United States	February 2021	For lithotripsy-enabled, low-pressure balloon dilatation of severely calcified, stenotic de novo coronary arteries prior to stenting.
Japan	March 2022	For disruption of severely calcified, de novo coronary lesions, allowing subsequent dilatation of a coronary artery stenosis.

IVL, intravascular lithotripsy

### Safety consideration

During the approval process for the IVL system, the applicant consulted with academic experts regarding training on the use of IVL. This device uses the same platform as balloon catheters for PCI and does not require new procedural techniques. According to its technical characteristics, nonclinical and clinical trial results, and overseas reports, no significant safety concerns or particular issues were identified; therefore, postmarketing surveillance was deemed unnecessary during the approval review. While the IVL system is not complicated to use, the appropriate indications for IVL can be difficult to understand if one has no sufficient knowledge of atherectomy and the role of imaging techniques. Therefore, the applicant collaborated with the Japanese Association of CVIT to provide a training program primarily focused on the criteria for the appropriate use of IVL. Additionally, for safety, the IVL system will be introduced into the clinical setting gradually, in accordance with the implementation plan.

## Guidelines for the proper use of IVL

The guidelines for proper IVL use developed by CVIT included eligibility criteria for medical institutions and physicians, proper use protocols, and treatment algorithms. The expert consensus document has also been published [19]. In developing the guidelines, CVIT considered the complexity and high risk involved when treating severe calcified lesions, and aimed to establish appropriate methods for using treatment devices, including IVL. The guidelines define a treatment algorithm based on imaging-based severity assessment and imaging passage, and clarify the use of different devices. They also do not recommend using IVL in combination with an atherectomy device or DCB because it was not verified in the Disrupt CAD III and Disrupt CAD IV studies. However, such guidelines will be revised if accumulated clinical evidence supports the use of such combination approaches in clinical practice. Therefore, the Dual-Prep Registry (jRCT1032230384) was planned for IVL treatment combined with atherectomy and MARBLE-J (jRCTs032240483) for IVL treatment combined with DCB.

## Revision of guidelines for proper IVL use by using real-world data

A drawback of IVL use is its limited ability to cross the lesion, especially the severely calcified ones. Thus, some lesions first require atherectomy so that the IVL system can pass through. In other cases, severe calcification remains after atherectomy passage, and the atherectomy size can be difficult to increase because of the high risk of complications. Guidewire bias can also be ineffective in some cases, making further size expansion unlikely to succeed. In such cases, the combination of atherectomy and IVL is considered as a complementary and synergistically effective treatment strategy. The use of atherectomy is more frequent in Japan than in other countries because calcified lesions are known to be highly frequent in dialysis, elderly, and diabetes mellitus cases. Therefore, the Dual-Prep Registry was conducted to confirm the safety and efficacy of IVL use after atherectomy [20]. This study included serial evaluations by OCT, and all patients had a calcification score of 4 even after atherectomy, indicating that atherectomy alone was inadequate; thus, additional treatment was necessary. According to the analysis of previously published, real-world atherectomy studies, at least 110 eligible patients are needed to achieve sufficient statistical power in assessing the primary safety endpoint. This sample size would enable evaluation of a 90% MACE-free rate with a 95% confidence interval of 82.4%–94.7% [3, 21–23]. In total, 118 patients with 120 lesions were enrolled at 20 sites. As the primary safety endpoint, the 30-day MACE-free rate was 98.3% (115/117; 95% CI, 94.0–99.8), and as the primary efficacy endpoint, the procedural success rate was 98.3% (116/118; 95% CI, 94.0–99.8). An independent Clinical Evaluation Committee evaluated all clinical events. Moreover, DES dilation was evaluated by OCT. The mean stent expansion index was 0.82, and 42.2% of the patients had a stent expansion index below 0.8. Anything below 80% suggests poor stent dilation, and the reported results of various devices for calcified lesions were all between 70% and 75% [3, 23–25]. Therefore, the Dual-Prep Registry suggested that IVL combined with atherectomy is effective.

**Fig. 1:**
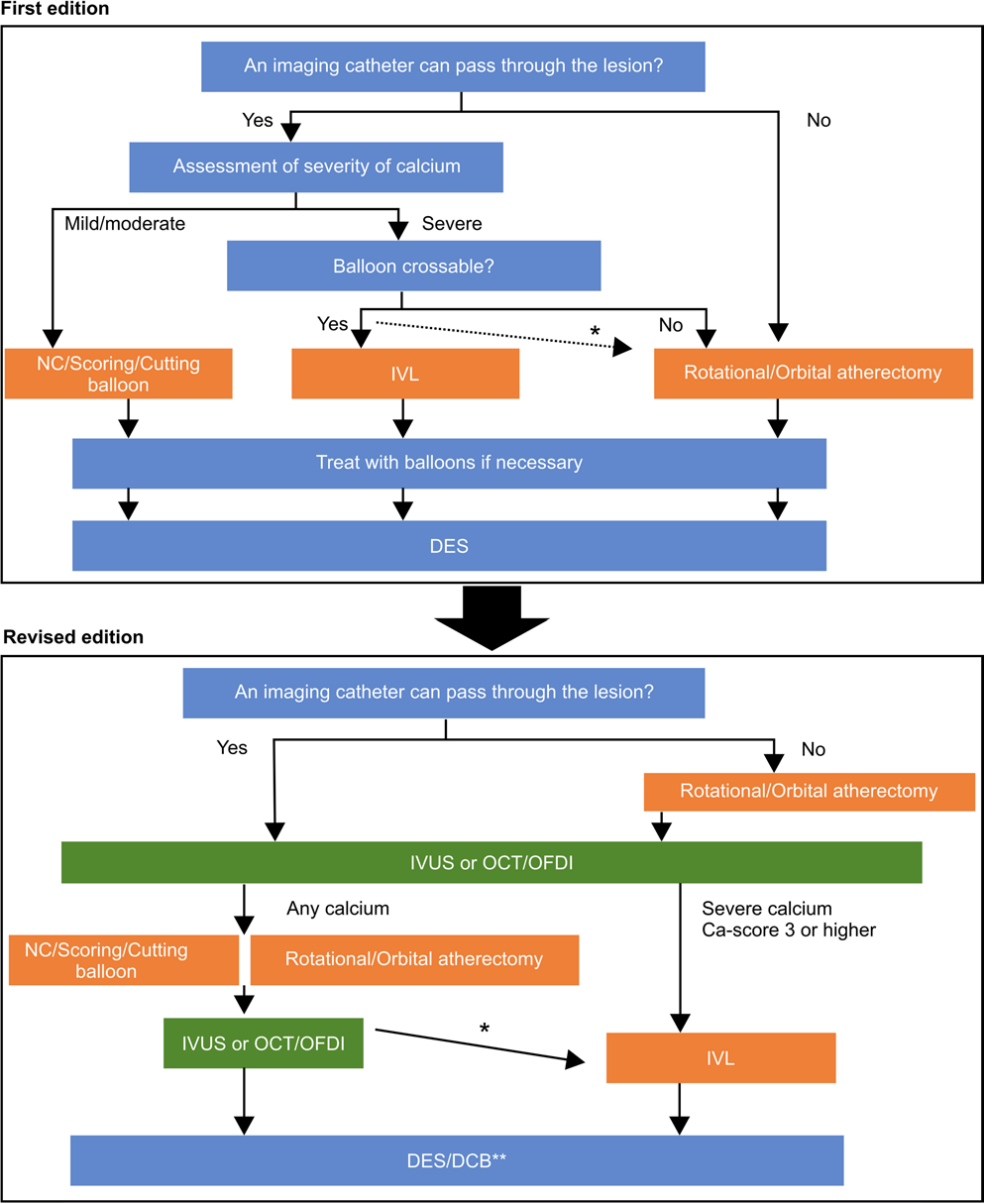
Revised strategy on device selection for calcified lesions, based on Dual-Prep Registry. Adapted from reference nos. 19 and 27 with permission. The top panel indicates the first edition [19], and the bottom panel indicates the revised edition [27]. DCB, drug-coated balloon; DES, drug-eluting stent; IVL, intravascular lithotripsy; IVUS, intravascular ultrasound; NC, noncompliant; OCT, optical coherence tomography; OFDI, optical frequency domain imaging

The conduct of the study was reported to the PMDA. Additionally, the independent consensus committee of CVIT evaluated the submitted analysis results and revised the guidelines for combining the use of the IVL system and the atherectomy device on a conditional basis [26]. The updated expert consensus document was also published (Fig. 1) [27].

## Discussion

This review outlines the approval process of the IVL system in Japan, the guidelines for proper use established by CVIT, and revisions based on the Dual-Prep Registry results. Given that the Dual-Prep Registry is a single-arm study, it cannot establish that this combination strategy is superior to other strategies. Therefore, this trial presents one treatment option for calcified lesions without demonstrating absolute significance. Essentially, this evidence applies not to all calcified lesions, but to those where calcification was considered an inadequate pretreatment, further atherectomy was inappropriate, or guidewire bias limited atherectomy’s effectiveness. This point was also explicitly stated in the consensus.

**Fig. 2:**
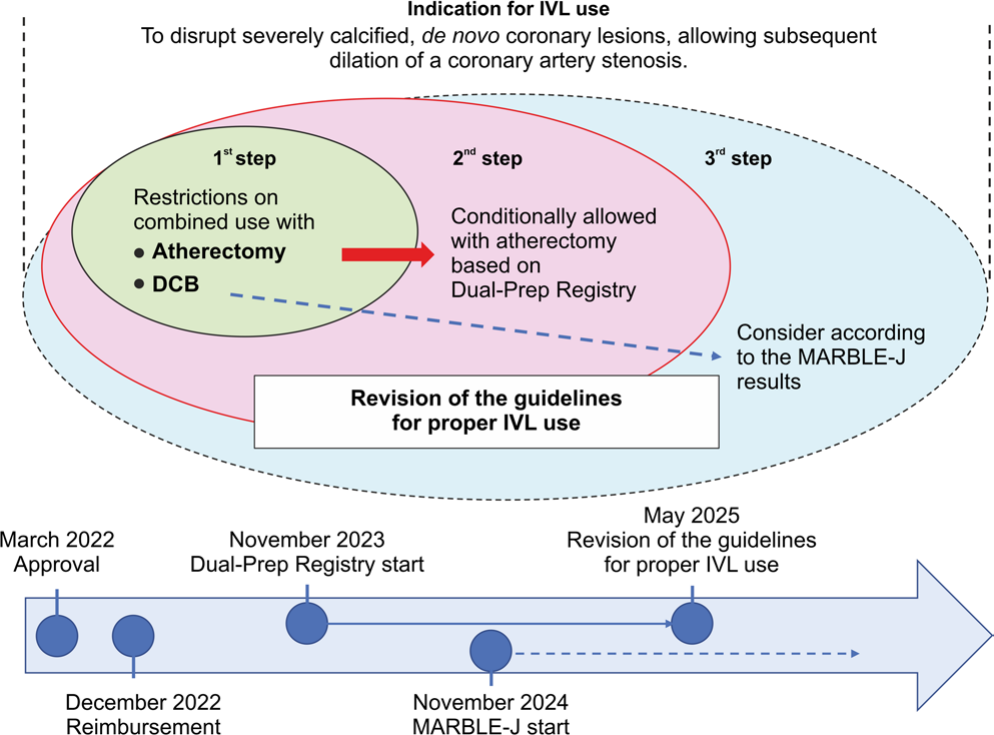
Phased introduction through revision of the guidelines for proper use. The initial guidelines for appropriate use (first step) did not recommend combining IVL with atherectomy and DCB. On the basis of the Dual-Prep Registry results, the guidelines were revised to allow the combined use of IVL and atherectomy (second step) [26]. Moving forward, a review of the guidelines based on the MARBLE-J results is planned as the third step. DCB, drug-coated balloon; IVL, intravascular lithotripsy

As mentioned above, concomitant use of IVL and DCB was not contraindicated during approval. However, the guidelines for proper use do not recommend it; evidence should be collected first and evaluated in clinical practice. The J-PCI registry indicates that the use of DCB has increased over time, reaching nearly 30% by 2023 [28]. Therefore, the MARBLE-J study has been conducted to investigate the combination of the IVL system and DCB, with plans to further revise the guidelines for their proper use in the future based on the results of this study. The results of this study are expected to inform future revisions of the guidelines for appropriate use (Fig. 2).

To the best of our knowledge, data on the comprehensive clinical impact of IVL (e.g., patient outcomes, procedural efficiency, and cost-effectiveness) in Japan are currently unavailable. However, through future J-PCI studies, data regarding its application in routine clinical practice and safety may be obtained. J-PCI seeks to evaluate the clinical impact of IVL and the degree of improvement in its appropriate use while also assessing the effectiveness of consensus revisions, thereby providing crucial information. Furthermore, the approach used in Japan to introduce IVL is unique and differs from that used in the United States and the EU. By comparing Japan’s regulatory framework, along with the clinical use of IVL and clinical evidence, with those of other countries, the effectiveness of this phased introduction will likely be discussed in detail.

Medical devices often undergo frequent improvements, and their indications often expand beyond the scope of premarket clinical trials. Hence, efforts to optimize treatment by supplementing limited premarket evaluations with postmarketing real-world data are gaining attention. However, given that regulatory approval is confined to the indications evaluated in clinical trials, real-world applications outside that scope are classified as off-label, hindering the accumulation of clinical data necessary for future indication expansion. Furthermore, conducting clinical trials to expand the range of indications requires an enormous amount of time and cost, indicating a major challenge in meeting the needs of the medical settings to expand the indication. If the indications are overly broad and include areas with insufficient evidence, patients may be highly at risk for more complications, considering that the efficacy and safety of these areas have not yet been confirmed.

**Fig. 3:**
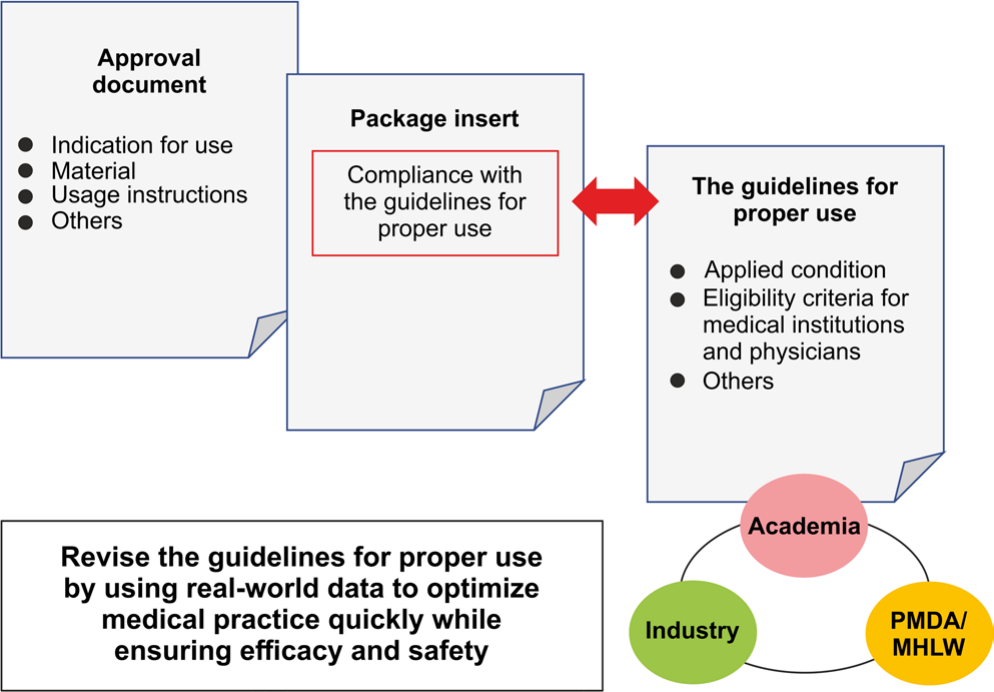
Ensuring safety by utilizing the guidelines for proper use and promptly reflecting them in clinical settings through evidence-based revisions. IVL, intravascular lithotripsy; MHLW, Ministry of Health, Labor, and Welfare; PMDA, Pharmaceuticals and Medical Devices Agency

To address these issues, the scope of intended use should be reviewed, including clinical trial results obtained during application for approval and other literature reports. The industry, academia, and government should also collaborate to ensure careful initial introduction in accordance with the guidelines for proper use and to revise such guidelines based on real-world data. This step may help ensure both safety and prompt implementation in clinical practice (Fig. 3).

In conclusion, this initiative is a unique approach in Japan and highlights the growing importance of collaboration among industry, academia, and government. Such efforts are expected to serve as a useful measure to ensure the prompt and safe introduction of innovative medical devices into medical practice in the future.
